# Recapitulation of Tumor Heterogeneity and Molecular Signatures in a 3D Brain Cancer Model with Decreased Sensitivity to Histone Deacetylase Inhibition

**DOI:** 10.1371/journal.pone.0052335

**Published:** 2012-12-18

**Authors:** Stuart J. Smith, Martin Wilson, Jennifer H. Ward, Cheryl V. Rahman, Andrew C. Peet, Donald C. Macarthur, Felicity R. A. J. Rose, Richard G. Grundy, Ruman Rahman

**Affiliations:** 1 Children’s Brain Tumour Research Centre, School of Clinical Sciences, University of Nottingham, Nottingham, United Kingdom; 2 Division of Reproductive and Child Health, School of Medicine, University of Birmingham, Birmingham, United Kingdom; 3 Division of Drug Delivery and Tissue Engineering, Centre for Biomolecular Sciences, School of Pharmacy, University of Nottingham, Nottingham, United Kingdom; 4 Department of Neurosurgery, Nottingham University Hospitals, Nottingham, United Kingdom; Instituto de Investigación Sanitaria INCLIVA, Spain

## Abstract

**Introduction:**

Physiologically relevant pre-clinical *ex vivo* models recapitulating CNS tumor micro-environmental complexity will aid development of biologically-targeted agents. We present comprehensive characterization of tumor aggregates generated using the 3D Rotary Cell Culture System (RCCS).

**Methods:**

CNS cancer cell lines were grown in conventional 2D cultures and the RCCS and comparison with a cohort of 53 pediatric high grade gliomas conducted by genome wide gene expression and microRNA arrays, coupled with immunohistochemistry, *ex vivo* magnetic resonance spectroscopy and drug sensitivity evaluation using the histone deacetylase inhibitor, Vorinostat.

**Results:**

Macroscopic RCCS aggregates recapitulated the heterogeneous morphology of brain tumors with a distinct proliferating rim, necrotic core and oxygen tension gradient. Gene expression and microRNA analyses revealed significant differences with 3D expression intermediate to 2D cultures and primary brain tumors. Metabolic profiling revealed differential profiles, with an increase in tumor specific metabolites in 3D. To evaluate the potential of the RCCS as a drug testing tool, we determined the efficacy of Vorinostat against aggregates of U87 and KNS42 glioblastoma cells. Both lines demonstrated markedly reduced sensitivity when assaying in 3D culture conditions compared to classical 2D drug screen approaches.

**Conclusions:**

Our comprehensive characterization demonstrates that 3D RCCS culture of high grade brain tumor cells has profound effects on the genetic, epigenetic and metabolic profiles of cultured cells, with these cells residing as an intermediate phenotype between that of 2D cultures and primary tumors. There is a discrepancy between 2D culture and tumor molecular profiles, and RCCS partially re-capitulates tissue specific features, allowing drug testing in a more relevant *ex vivo* system.

## Introduction

Brain tumors are the leading cause of cancer related death in children and adults up to the age of 40, accounting for 10,000 lost years of expected life annually in the UK. High grade invasive brain malignancies recur despite multimodal therapy; hence there is an urgent need to develop new treatment strategies to improve outcome. The imbalance in compound efficacies between *in vitro* and *in vivo* experiments has considerably hampered the discovery and development of a more effective and diverse armament of chemotherapeutic agents [1]. As a result, only ∼5% of cancer drug candidates that enter clinical trials will ever receive approval from the U.S. Food and Drug Administration, representing a huge financial and clinical burden [2], [3]. The lack of pediatric brain tumor models in particular, has meant that chemotherapy drugs that are currently prescribed had been initially developed for the treatment of adult central nervous system (CNS) and other solid tumors. This ignores accumulating evidence that adult and pediatric brain tumors, although histologically similar are molecularly distinct [4] and hence harbor distinct oncogenic mutations that may provide novel therapeutic targets. As next-generation therapy moves towards specific biological and pathway-related targets, evaluation of drug efficacy and improved treatment will be achieved not only via novel drug discovery, but also through improved pre-clinical *ex vivo* models that better recapitulate micro-environmental complexity [5]. There is growing appreciation of the intratumoral heterogeneity of high grade brain tumors with the existence of differing cell populations within the same tumor that each display different molecular changes [6]. Current pre-clinical drug testing models do not replicate these key features.

Two-dimensional (2D) monolayer cell cultures represent a highly reductionist model of cancers due to the loss of physiological extracellular matrix (ECM) on artificial plastic surfaces. Consequently, cells under these conditions adapt by altering gene expression patterns and signaling networks, lose relevant properties such as differentiation, polarization and cell-cell communication, and artificially promote hyper-proliferation [1], [7]. The ease of drug uptake into a homogenous cell population on adherent 2D surfaces, also likely results in an overestimation of efficacy determined from *in vitro* cytotoxic screens, leading to ineffectiveness of candidate agents *in vivo* [8]. Moreover, 2D cultures lack complex 3D tissue structure and therefore hamper efforts to test the effectiveness of certain candidate compounds such as anti-angiogenic/vasculogenic agents prior to *in vivo* strategies [8]. Indeed 2D cytotoxic screens almost exclusively identify anti-proliferative candidate agents [9], [10].

There is a growing recognition conceptually that three-dimensional (3D) culture technologies may more accurately reflect tumor pathophysiology and recapitulate the *in vivo* tumor microenvironment *in vitro*. As pharmacological responses in xenograft studies do not consistently correlate with the clinical response, it is imperative that better human-specific predictive models are developed at a pre-clinical stage. Consequently, these models should better inform xenograft studies and early clinical trials in terms of predicting the pharmacological response to chemotherapeutic compounds [11], [12], [13] thereby additionally reducing the requirement of substantial numbers of animals for anti-cancer drug testing.

Multicellular spheroids are small *in vitro* aggregates ranging from 100–1000 µm in diameter and commonly cultured in suspension using spinner flasks, collagen gels and liquid-overlays [14]. The size of the cultured spheroid is limited to less than 1 mm by shear forces in the culture system and lack of internal cohesion/ECM within the spheroid. The geometry of the spheroid contributes to the formation of heterogeneous cell populations: the outer layer proliferates comparably to monolayer cultures; the inner layer of cells can become quiescent; and the core of spheroids can develop necrosis [15]. The proliferation of tumor cells grown in 3D spheroids is typically slower than that of monolayer cultures, exhibits different metabolic profiles and typically displays reduced sensitivity to chemotherapy and radiotherapy [7], [16], [17], [18], [19], [20]. For example, cytosine arabinoside (Ara-C) and Taxol which inhibit proliferation of glioblastoma cell lines in 2D cultures, but have failed clinical trials, show markedly reduced sensitivity in 3D cultures; an Ara-C dose 10 times higher than the effective dose in 2D cultures was necessary to reduce tumor cell proliferation [21], [22], [23]. A more recent 3D spheroid culture system from the Eccles and colleagues has provided automated, quantitative imaging capability that is potentially compatible with high-throughput preclinical targeting studies and may reduce the requirement of animal studies. Moreover functional assays of tumor migration and invasion are permitted in this system, thus facilitating drug screening that covers a broad range of compounds [24]. Consistently, gene expression signatures of primary cell cultures are maintained in spheroid cultures such as glioblastoma [25], [26].

Biological gels such as Matrigel have also been used as a substrate for spheroid growth. Matrigel is a basement membrane extract derived from the Engelbreth-Holm-Swarm mouse sarcoma that contains a diverse array of components, including collagen type IV, laminin and other ECM molecules, in addition to soluble signals such as cytokines and growth factors [27]. LaBarbera and colleagues demonstrated that tumor-related phenomena such as epithelial-mesenchymal transition in metastatic breast tumors can be recapitulated in 3D matrigel-based spheroid culture, a system additionally used as a high-throughput screening tool for small molecule inhibitors for breast carcinoma [28]. However, as Matrigel is a largely undefined and variable mixture of proteins, it has not been used widely for drug screening purposes. Porous collagen/hyaluronic scaffolds provide a more physiologically relevant *in vitro* model that permits the interactions between different cell types and ECM as described recently for mammary epithelial and adipocyte co-cultures [29]. Other breast cancerous 3D models have been described recently in which malignant breast cancer cells are embedded in reconstituted basement membrane with direct co-culture of endothelial cells, enabling investigation of the stimulatory effects of the latter cell type [30]. Suspension cultures such as spinner flasks have been investigated but are often hindered by shear stress and turbulence [31] effects experienced by the cells.

One method of generating 3D aggregates in a non-turbulent environment and where cells secrete endogenous ECM and growth factors is the Rotary Cell Culture System (RCCS™), originally developed by the National Aeronautics and Space Administration (NASA) to study the effects of microgravity on cells and tissues [32]. The system has been successfully used in tissue engineering [33], developmental biology [34] and microbiology [35]. The RCCS method has been extended to tumor cell propagation (e.g. in prostate cancer [36] and colorectal cancer [37]) where minimal shear force and continually suspended ‘free-fall’ culture encourages cell aggregation. This permits biological/biochemical processes to develop under closely monitored environmental and operational conditions and encourages cells to form 3D tissue-like aggregates which display physiologically relevant phenotypes such as lower proliferation rate, hypoxic regions and vasculature [1]. No previous publications have specifically examined brain tumor growth in the RCCS or made comparison between 2D culture, 3D culture and actual brain tumor specimens. Analysis of gene expression, microRNA and metabolic profiles demonstrates that our RCCS aggregates (in comparison to 2D monolayer cultures) display a genotype significantly more similar to actual brain tumors and may thus replicate tumor behavior (e.g. response to therapeutic agents) more faithfully. In addition, 3D aggregates can be cultured for at least up to 4 weeks, compared to approximately one week in 2D monolayer culture (due to cell confluency), providing a means to assess drug efficacy and repeated dosing over a longer time period. No artificial matrix or ECM components are introduced, allowing cells to produce their own structures and with no artificial barriers to drug diffusion. The increased size and heterogeneity of RCCS aggregates compared to other culture methods e.g. neurospheres or multicellular spheroids, increases their physiological relevance, allowing drug testing against a simulation of an entire tumor rather one selected cell line sub-clone as in 2D or neurospheres. Critically, the RCCS aggregates display marked internal heterogeneity with separate populations of proliferative, senescent and necrotic cells, influenced in part by the changing oxygen concentration from rim to core of the aggregate. This uniquely recapitulates the variety of *in vivo* environmental niches within which cancer cells reside and which are now thought critical to tumor development and progression.

In the present study, we sought to compare neoplastic histology, morphology, proliferation rates, gene and microRNA expression, metabolic profiles and histone deacetylase inhibitor sensitivity between 2D monolayer cultures and 3D tumor cell aggregates generated using the RCCS™, in order to evaluate their relevance for basic brain cancer disease modeling and pre-clinical drug discovery. To our knowledge, this is the first comprehensive molecular characterization of brain tumor aggregates cultured using the RCCS, demonstrating considerable similarity in the expression levels of key neoplastic genes to actual tumors and the first report of RCCS drug sensitivity in comparison to 2D cultures.

## Materials and Methods

### Cell Lines – two and Three Dimensional Culture

The following established cell lines were utilized in the study – KNS42 (pediatric GBM, a kind gift from C. Jones, Institute of Cancer Research, London [38], [39]), U87MG (adult GBM) [40], [41], PFSK-1 (pediatric central nervous system primitive neuro-ectodermal tumor, purchased from ATCC [42]), DAOY (pediatric medulloblastoma, purchased from ATCC [42], [43]), GB1 (in-house derived pediatric high grade glioma [42]), SF188 (pediatric glioblastoma, [44], [45]) and HBMEC (human brain microvascular endothelial cell, a kind gift from Naveed Khan, University of Nottingham, [46], [47]). KNS42 were maintained in Dulbecco’s modified Eagle’s medium (DMEM)/F12, U87MG, DAOY, C6 and GB1 in DMEM, PFSK-1 and HBMEC in Roswell Park Memorial Institute (RPMI) medium 1640. All media were supplemented with 10% (*v/v*) fetal bovine serum (except HBMEC which were in 20% (*v/v*) fetal bovine serum), 100 IU/ml penicillin and 100 µg/ml streptomycin. The same media were used for each cell line in 2D and 3D culture and all culture took place in a humidified incubator at 37°C and 5% CO_2_ atmosphere, except the hypoxic controls for the Hypoxyprobe study which were undertaken in a hypoxic chamber in a 1% O_2_ environment. 3D culture utilized the Rotary Cell Culture System (RCCS) (Synthecon, Luxembourg) situated within a cell culture incubator. Culture was commenced by introducing 1×10^6^ cells as a single cell suspension into a 10 ml culture vessel and initially rotated at 15 revolutions per minute (rpm). Once a visible aggregate formed, revolution speed was adjusted to balance the Coriolis force against gravity to maintain the aggregate in stationary freefall. Aggregates were harvested at three weeks with media being changed twice per week (50∶50). Once harvested, aggregates were either fixed in 4% (*w/v*) PFA or stored frozen at −80°C until required for further analysis.

### Preparation of RNA and Real Time PCR

Total RNA was isolated from cell pellets or aggregates using the *mir*Vana RNA isolation kit (Ambion, Austin,Tx). Reverse transcription to cDNA (including genomic DNA elimination) was performed using the RT^2^ First Strand Kit (SA Biosciences, Frederick, MD). Array real time PCR was performed using the SA Biosciences extracellular matrix and adhesion molecules PCR array (PAHS-013A), analyzing 84 ECM related genes, 5 control genes and positive and negative control wells (Table S1), using a CFX96 real-time PCR machine (BioRad, Hercules, CA). Validation real-time PCR for the genome wide data was performed against the genes listed in Table S2. Primer efficiency was calculated and the expression of each gene calculated relative to normal adult brain RNA (FirstChoice, Ambion) using the modified Pfaffl equation R =  E_target_ ΔCT (CT target gene control-CT target gene sample)/E_control_ ΔCT (CT control gene control- CT control gene sample) with GAPDH as the control gene.

### Patients and Tissue Samples

The cohort of patient samples (n = 53) analyzed in the gene expression array were a group previously published [4] with data available online (GEO accession number GSE19578). All samples were *de novo* pediatric high grade gliomas obtained *ante mortem* in UK Neurosurgical centers with all diagnoses confirmed by central pathological review. The cohort of samples analyzed for microRNA expression (n = 77, 72 individuals) consisted of 21 samples also analyzed in the gene expression cohort, with the other samples also reviewed centrally and all of primary pediatric high grade gliomas. Full consent and ethical approval has been obtained for their use in this study, from the U.K. Children’s Cancer and Leukaemia Group and local ethical and Trent MREC approval (06/MRE04/86).

### Gene Expression Array and MicroRNA Array

Gene expression analysis was performed on the Affymetrix U133 plus2 platform, with triplicate experimental repeats for the 3D cultures. MicroRNA analysis was performed using the Nanostring platform (Nanostring Technologies, Seattle, WA) against 747 human and viral microRNA species (complete list of probes and targets in Table S3).

### Magnetic Resonance Spectroscopy (MRS)

Three week tumor aggregates were harvested, washed with PBS and flash frozen in liquid nitrogen ensuring no trace of PBS or media remained. Prior to NMR analysis, cell aggregates were thawed at room temperature before being placed into 4 mm zirconia rotors with 12 or 50 µl inserts depending on sample volume. Any remaining volume in the rotor was filled with D_2_O containing a trace of trimethylsilylproprionate-d4 to aid chemical shift calibration. NMR analysis was performed on a Bruker HCD hr-MAS probe with pulsed gradients available in the z-direction. The probe operated at a magnetic field strength of 500 MHz generated by an actively shielded Oxford Instruments magnet with a 3-channel Bruker Avance II console. Sample spinning was set at a rate of 4800 Hz and probe temperature was set to 278 Kelvin to minimize metabolic activity. The NOESY-presat sequence was used to suppress the water signal and 16K data points were acquired at a spectral width of 16ppm following a 90 degree pulse. Signal averages (256 or 512) were acquired, depending on the signal to noise ratio of the sample, taking 14 or 28 minutes respectively. Raw NMR data was processed using the TARQUIN algorithm [48] to extract quantities for the following metabolites: acetate (Ace); alanine (Ala); choline (Cho); creatine (Cr); glutathione (Glth); glutamine (Gln); glutamate (Glu); glycine (Gly); guanidinoacetate (Gua); glycerophosphocholine (GPC); hypotaurine (h-Tau); myo-inositol (m-Ins); lactate (Lac); lipids (Lip); n-acetylaspartate (NAA); phosphocholine (PCh); scyllo-inositol (s-Ins); succinate (Suc) and taurine (Tau). Lac was removed from subsequent analysis due to its high dependence on cell culture media withdrawal. Metabolite profiles were normalized to their sum and quantities were scaled to give equal variance. A heatmap plot was generated with dendrograms on both axes calculated using the complete linkage method.

### Drug Testing and Alamar Blue Viability Assay

For 2D monolayer proliferation assays, U87 and KNS42 cells were seeded onto wells of a 24-well plate at a density of 5×10^4^ cells per well. Alamar Blue proliferation assay was first conducted 24 hours post-seeding (designated Day 1) and conducted on three successive days subsequently. Briefly, media was removed from the wells and cells washed twice with PBS to ensure no trace of phenol red remained. Fresh PBS (1 ml) was added to each well containing tumor cells followed by the addition of 100 µl Alamar Blue indicator dye (Invitrogen, UK). Following incubation for 2 hours at 37°C, 100 µl aliquots were transferred to single wells of a 96-well black-bottom plate in triplicate and fluorescence emission measured at 585 nm using a plate reader (Tecan, Switzerland). Wells containing 2D cells were replaced with fresh media and this procedure repeated on Days 2, 3 and 4 in order to monitor proliferation of each cell population over three days. For 3D culture proliferation assays, U87 and KNS42 cells were cultured as RCCS aggregates at 10–15 rpm for 1 week. The following day was designated Day 1 of proliferation analysis and all subsequent proliferation readings (arbitrary fluorescence units) were calculated relative to the Day 1 baseline reading, thus accounting for variations in aggregate size after 1 week culture. 3D aggregates were removed from the RCCS vessels and placed in single wells of a 24-well tissue culture plate and washed twice with PBS. As above, 1 ml of fresh PBS was added to each well containing an aggregate followed by the addition of 100 µl Alamar Blue indicator dye (Invitrogen, UK). Following incubation for 2 hours at 37°C, 100 µl aliquots were transferred to single wells of a 96-well black-bottom plate in triplicate and fluorescence emission measured at 585 nm using a plate reader (Tecan, Switzerland). Tumor aggregates were subsequently returned to UV-irradiated RCCS vessels and 3D culture resumed. This procedure was repeated on Days 2, 3 and 4 in order to monitor real-time proliferation of aggregates over three days. Data for all proliferation analyses is presented as the average percentage cell viability of three independent experiments with error bars indicating standard error of the mean (SEM). For cytotoxicity analyses of 2D monolayers, cells were seeded 24 hours prior to drug treatment at a concentration range of 0–10 µM and the Alamar Blue assay conducted after 72 hours drug exposure. For cytotoxicity analyses of 3D cultures, cells were grown in the RCCS for 1 week to allow aggregates to form, followed by removal of the aggregates from the RCCS vessels and placing in single wells of a 24-well plate. The Alamar Blue assay was conducted as previously described and proliferation status at this stage designated as baseline. Aggregates were returned to UV-sterilized RCCS vessels and cultured in media containing Vorinostat (Selleck Chemicals, 5 mM stock) at a concentration range of 0–30 µM. Proliferation analyses were repeated after 72 hours drug exposure and data normalized to baseline readings to take into account varied aggregate sizes due to stochastic variation in the RCCS 3D culture.

### Immunohistochemistry

Immunohistochemistry was performed according to previously published protocols [49]. Aggregates were formalin fixed and wax embedded prior to sectioning. Antigen retrieval was conducted by steaming in citrate buffer for 40 minutes. Anti-Ki67 (Dako, monoclonal mouse anti-human Ki67 antigen, clone MiB-1) was applied for 30 minutes at room temperature at 1∶50 concentration. Anti-beta galactosidase (Abcam, mouse monoclonal DC1 4C7) and anti-CDKN2A/p16INK4a (Abcam, mouse monoclonal 2D9A12) were utilized at 1∶1000 and 1∶800 concentrations respectively for one hour at room temperature. Secondary antibody (100 µl Dako horse radish peroxidase conjugated rabbit anti-mouse) was applied for thirty minutes at room temperature before application of 3,3′-Diaminobenzidine chromogen. Positive control tissues were tonsil (Ki67) and colon carcinoma (beta-galactosidase and p16). Immunohistochemistry for Ki67, p16 and beta-galactosidase was scored by counting positive nuclei as a percentage of total nuclei within multiple high powered fields (at least three for each specimen). For tumor specimens on the tissue microarrays, the most positive area (‘hot-spot’) for each core was scored. For cell cultures undergoing hypoxyprobe (Hypoxyprobe Inc., Burlington, MA) analysis, the hypoxyprobe reagent (pimonidazole) was applied two hours before harvesting at 200 µM concentration. Samples were then fixed as standard and immunohistochemistry performed using the primary antibody supplied with the hypoxyprobe kit at 1∶200 concentration. Positive controls were performed using 2D cell cultures cultured in a hypoxic chamber at 1% atmospheric oxygen. Negative controls were performed by omitting the primary antibody and also with monolayer cells cultured in 21% oxygen, 5% CO_2_ atmospheric conditions.

### Immunofluorescence

Monolayer cells or 3D RCCS aggregates were fixed in 0.4% paraformaldehyde and blocked in 5% normal goat serum/0.1% Triton-X for 1 hour at room temperature. For immunolabelling, cells were incubated with anti-beta galactosidase (Abcam, mouse monoclonal DC1 4C7) using a 1∶1000 dilution overnight at 4°C in a humidified chamber. Cells were washed and incubated with the anti-mouse Alexa-fluor 555 (Dako) using a 1∶300 dilution in the dark at room temperature. Nuclei were visualized using DAPI and fluorescence signals were recorded using a Leica DMRB upright fluorescent microscope.

### Scanning Electron Microscopy

Three dimensional tumor aggregates were washed three times in fresh PBS and fixed in 3% glutaraldehyde for 12 hours. Fixed samples were then washed three times in PBS and dehydrated for a further 12 hours by exposure to air at room temperature. Fixed and dehydrated aggregates were mounted on aluminum stubs and were sputter-coated with gold at an argon current rate of 30 mA for 3 minutes. Cell morphology and organization in the aggregates was visualized using a scanning electron microscope (SEM) (JEOL JSM-6060LV) at 10 kV.

### Biostatistical Analysis

Analysis of gene expression and microRNA array data was conducted using the Genespring package (Agilent, UK) with comparison between groups performed using unpaired two-tailed t-test with Benjamini-Hochberg multiple test correction. Analysis of the array PCR data was undertaken using the accompanying Excel based statistical package (SABiosciences). Pathway analysis was performed using the Ingenuity IPA package (Ingenuity systems). SPSS software was used to conduct student t-test and P values were considered significant at less than 0.05 throughout.

## Results

### Macroscopic 3D RCCS Aggregates Recapitulate the Heterogeneous Morphology of Primary Brain Tumors

Visible macroscopic cell aggregates were observed after 24–48 hours of initiating culture and achieved maximal size after one week of culture. Diameter of aggregates was broadly consistent for each cell line utilized but varied between different cell lines. The largest aggregates resulted when using the PFSK-1 cell line at up to 9 mm diameter and KNS42 (up to 5 mm). U87 formed multiple (5–10) smaller aggregates each 1–3 mm in diameter. Cultured aggregates were maintained viable in culture for up to six weeks, but were typically harvested at three weeks for molecular and cellular characterization.

**Figure 1:**
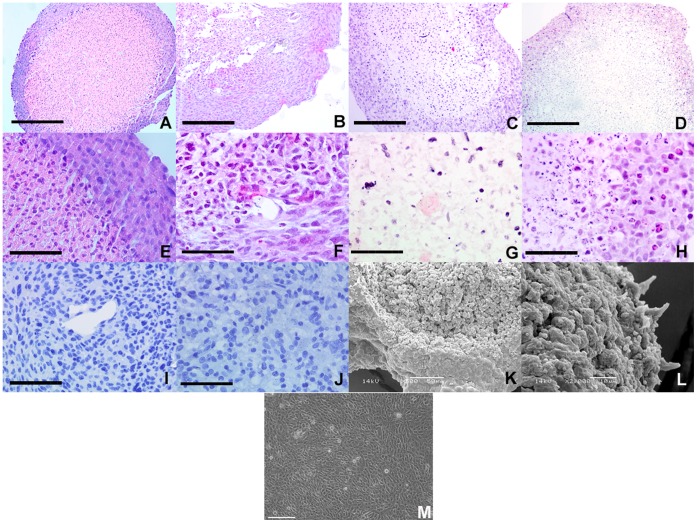
Morphology and cellular heterogeneity of RCCS aggregates. **A–D** –Low power views of sections of formalin fixed paraffin embedded GB1, U87, DAOY and PFSK-1 aggregates respectively, demonstrating low cellularity necrotic core and densely cellular proliferative rim. **E–H –** High power views of GB1, U87, KNS42 and PFSK-1 respectively, demonstrating cellular heterogeneity at the rim/core interface and necrotic tissue with apoptotic figures in the core. **I&J –** GB1 aggregate and sample of primary tumor from which cell line was derived respectively demonstrating similarity in cellular morphology and growth density. **K&L –** Scanning electron microscopy of GB-1 RCCS aggregates with dense rim and exposed core in K and high powered surface view in L. M – Morphology of GB-1 2D monolayer cells. Scale bars 100 µm in A–D and 25 µm in E–J and M. Magnification×500 in K and ×2000 in L.

Aggregates from all cell lines had similar overall reproducible morphology with a highly cellular rim (typically 100–200 µm thick) of viable cells, an intermediate transitional zone of mixed healthy and dying cells and a central core of low cellularity with the majority of cells necrotic or apoptotic (Figure 1 A–H). For the derived GB-1 cell line, comparison was possible with the original primary tumor and this demonstrated very similar morphology and cellular growth density with a mean of 424 cells per high powered field (hpf) for the primary tumor and 398 cells per hpf for the 3D aggregate (no statistically significant difference, p = 0.452) (Figure 1 I–J). The 3D aggregates also resembled tumors in their re-capitulation of heterogeneity with high and low cellularity regions. The high cellularity rim and low cellularity core was also visualized with scanning electron microscopy with secreted extracellular matrix elements observed as the 3D aggregates began to lay down endogenous ECM (Figure 1 K–L). In contrast, GB-1 2D monolayer cells display a more homogeneous morphology throughout the culture vessel with no distinct regions spatially (Figure 1M).

### RCCS Aggregates Display Cellular Heterogeneity and Reduced Proliferation Rates

**Figure 2:**
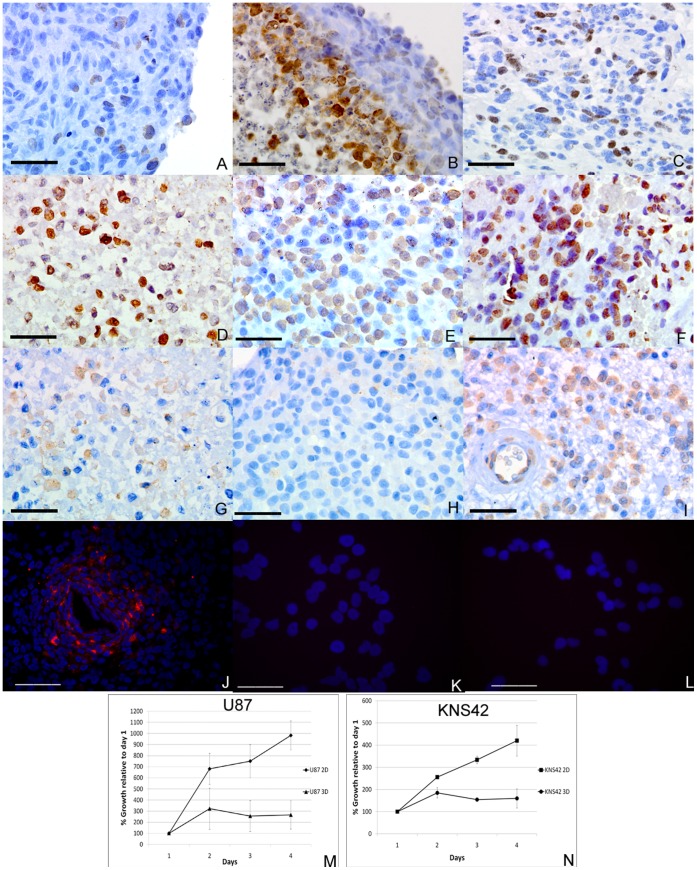
Heterogeneous cellular phenotypes and reduced proliferation rates of RCCS aggregates. **A–C** –Immunohistochemistry against Ki67 for U87, PFSK-1 and primary HGG respectively demonstrates proliferative cells in the rim and/or rim/core interface of aggregates at similar frequencies to primary tumor. **D–F –** Immunohistochemistry against P16 in a KNS42 aggregate core (D), KNS42 rim (E) and in a primary HGG (F) showing a higher proportion of cells staining positive in the aggregate core compared to the rim and which is comparable to the primary tumor. **G–I –** Immunohistochemistry against beta-galactosidase in a KNS42 aggregate core (G), KNS42 aggregate rim (H) and in a primary HGG (I) showing that significant staining for senescence markers occurs in both resected tumors and aggregate cores. **J–L –** Immunofluorescence against beta-galactosidase in KNS42 aggregates, KNS42 2D monolayer cells and U87 2D monolayer cells. **M&N –** Alamar Blue assay proliferation rates in U87 (J) and KNS42 (K) comparing 2D and RCCS culture demonstrating significantly decreased proliferation in 3D compared to 2D for both cell lines. Scale bars in A–I and K–L are 25 µm. Scale bar in J is 100 µm.

Immunohistochemistry for Ki67 revealed the great majority of proliferative cells in 3D aggregates to be within the rim or transitional zone, with no or very few positive cells in the central core (Figure 2 A–C). The exact pattern of staining varied slightly between cell lines but all lines exhibited these general characteristics. Staining for p16 and beta-galactosidase, markers of cellular senescence, exhibited similar results, whereby both antibodies stained a much higher proportion of cells within the core as compared to the rim. For the KNS42 line, P16 stained 90% of cells in the core and 40% of cells in the rim, whereas beta-galactosidase stained 47% in the core and 1% in the rim. This pattern was repeated for all the other cell lines (data not shown). Both P16 and beta-galactosidase showed a similar proportion of positive staining in the core of primary tumors compared to the core of 3D aggregates (Figure 2 D–I). Immunohistochemistry for these targets was also performed against our cohort (n = 150) of pediatric high grade glioma archival specimens on tissue microarrays. We found a statistically significant association between Ki67 staining in perivascular areas and patient survival as previously reported [49]. No significant correlation was found between p16 staining and survival (p = 0.577 Cox regression) and only a weak trend to better survival with elevated numbers of cells positive for beta-galactosidase (p = 0.161 Cox regression, p = 0.09 Logrank). Interestingly, cells that were positive for senescence markers (typically regarded as a tumor specific characteristic) were observed in the vessel wall and even within the endothelial lining (Figure 2I). Beta-galactosidase staining indicative of cellular senescence was clearly defined around the peri-necrotic region of KNS42 aggregates (Figure 2J) whereas no positive staining was evident in either KNS42 or U87 2D monolayers (Figure 2K–L). Proliferation assays demonstrated significantly reduced rates of proliferation in 3D cultures of KNS42 and U87 compared to the same cell lines cultured in 2D monolayers (p<0.05) (Figure 2 M–N). Relative growth over three days in 3D cultures was 27% and 38% of the 2D growth rates for U87 (population growth from day 0 (100%) was 266% vs. 982%) and KNS42 (population growth from day 0 (100%) was 159% vs. 420%) respectively.

**Figure 3:**
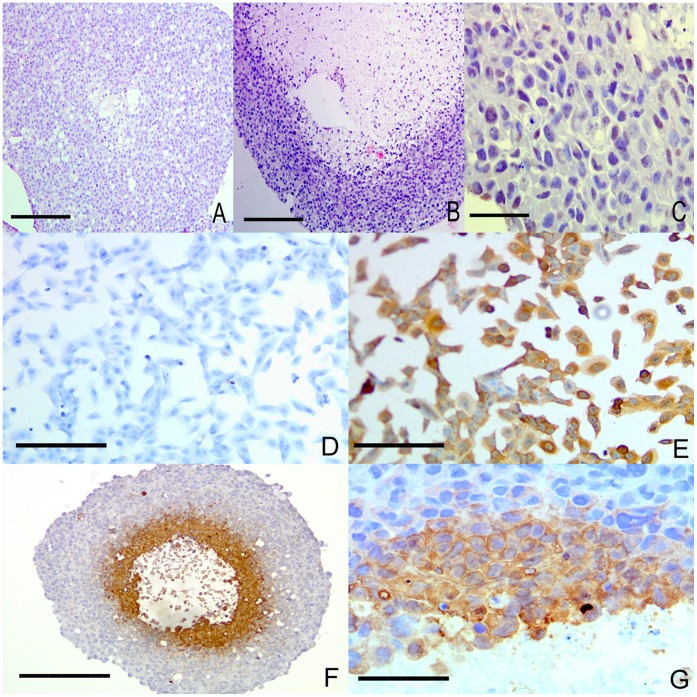
RCCS aggregates exhibit heterogeneous oxygen tension. **A –** Co-culture in RCCS of KNS42 and HBMEC brain endothelial cells demonstrating a reduced necrotic core compared to pure KNS42 culture (**B**). **C –** High powered view of rim of co-culture stained positive for von Willebrand factor with mixed cell morphologies. **D –** KNS42 2D monolayer culture grown with hypoxyprobe in normoxia (21%) with no positive staining. **E –** KNS42 2D monolayer culture grown with hypoxyprobe in 1% oxygen showing widespread positive staining. **F –** U87 aggregate cultured in RCCS (21% oxygen) demonstrating positive staining in deep hypoxic rim with no staining in normoxic peripheral rim or in fully necrotic core. **G –** KNS42 aggregate showing positive staining in cells deep within the aggregate rim adjacent to the necrotic area. Scale bars 100 µm in A, B and F; 25 µm in C, D, E and G.

Co-culture of HBMEC brain endothelial cells with KNS42 cells had marked effects on aggregate cellular morphology (Figure 3 A and C). The necrotic core of aggregates was substantially reduced in size compared to monoculture of the KNS42 line alone, with numerous tubular structures also apparent. Cells positive for the endothelial marker von Willebrand factor (vWF) were intermingled with negative cells in the viable rim area. Hypoxyprobe staining demonstrated consistent and specific staining on 2D monolayer cells cultured under hypoxia (1% oxygen atmosphere). The hypoxyprobe system utilizes cellular metabolism to produce the pimonidazole molecule targeted by immunohistochemistry, where only viable cells stain positive. Dead or dying cells cannot produce the active metabolite and thus remain unstained, although the oxygen tension may be low in that area. On staining of 3D aggregates, a band of staining in the transitional zone between rim and core was observed, suggesting this as a region where the cells were under hypoxic strain but still viable (Figure 3 D–G). The cores were generally negative, suggesting cells here were either dead or unable to metabolize normally.

### Microenvironment of RCCS Aggregates Consists of Upregulated Endogenous ECM

**Figure 4:**
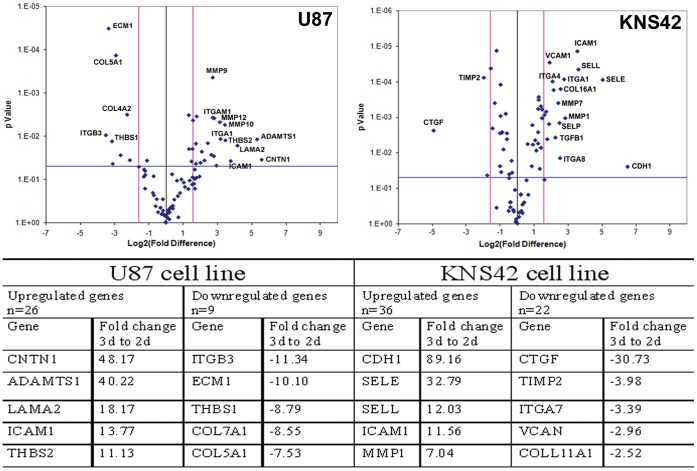
Upregulation of endogenous ECM in RCCS aggregates. Volcano plot of 84 ECM associated genes comparing expression after culture in 2D or 3D with upregulation in 3D shown as a positive fold change. Light grey vertical lines represent a fold change of +/−3 fold and the light grey horizontal line represents a p-value of 0.05. Selected significantly differentially expressed genes are labeled. Table lists top 5 (by fold change) significantly up- and down-regulated genes for each cell line. Three independent aggregates were used for each cell line and three ECM PCR arrays were conducted for each aggregate.

Analysis of 84 ECM related genes by array real-time PCR demonstrated significant changes in the expression levels of many genes. The differential patterns of expression between the same cell lines cultured in 2D and 3D are illustrated in Figure 4. Genes significantly upregulated in 3D culture included matrix metalloproteinases, transforming growth factor beta (TGF-β), cell adhesion molecules (CAM) and integrins. Genes downregulated included collagen subtypes, tissue inhibitors of metalloproteinases (TIMPs) and thrombospondin 1. A full list of differentially expressed genes is provided in Table S4. Immunohistochemistry was used to validate the upregulated expression of ICAM-1 in 3D relative to 2D culture (Figure S1).

### Genome Wide Expression in RCCS Aggregates is Intermediate to 2D Cultures and Primary Tumors

Genome wide gene expression analysis revealed significant numbers differentially expressed transcripts between 2D monolayer and 3D RCCS cultures. Comparison between 3D and 2D culture of the U87 adult GBM line demonstrated 19365 probes significantly differentially expressed (10159 probes upregulated and 9206 probes downregulated in 3D compared to 2D) accounting for 13116 unique gene expression profiles. The top 20 differentially expressed genes by fold change are listed in Table 1 with a full list of differentially expressed probes in Table S5. Transcripts with highest differential expression in U87 included several genes proposed to be involved with stem cell self renewal and differentiation, e.g. SOX2 and growth factor pathway genes, e.g. EREG and NEDD9. For KNS42 1771 probes were differentially expressed (969 upregulated and 802 downregulated in 3D compared to 2D), representing 1386 unique gene expression profiles. The most differentially expressed genes for KNS42 are also listed in Table 1 with a full list of differentially expressed probes in Table S6. Highly differentially expressed genes for KNS42 included multiple chemokines, interleukins and ECM modifiers. Genes highly differentially expressed in a similar fashion in both U87 and KNS42 included KYNU, IL1B and CXCL12.

**Table 1:** Top twenty genes by fold change differentially expressed between 2D and RCCS culture.

U87			KNS42		
Gene	Fold change 3d vs. 2d	Correctedp value	Gene	Fold change 3d vs. 2d	Corrected p value
GPM6B	572.66	1.13E−09	CCL20	619.68	0.005
IGFBP5	433.33	2.57E−10	IL8	210.72	0.006
SOX2	409.13	4.19E−12	PTGS2	88.61	0.010
SOX2OT	372.92	1.89E−11	SOD2	72.32	0.004
PTPRZ1	353.69	4.91E−10	EREG	70.47	0.010
TFPI2	−351.24	3.35E−10	IL1B	65.92	0.005
NEDD9	340.64	1.09E−11	CHI3L1	64.71	0.012
EPHA3	326.63	4.82E−14	TNFAIP6	32.12	0.008
MGP	304.67	4.36E−07	CXCL2	31.69	0.005
TOX3	299.67	7.16E−14	MMP1	29.71	0.032
GPM6A	272.30	4.57E−08	IL1A	24.40	0.005
CNR1	243.90	8.15E−10	FLG	−23.13	0.011
NID1	208.49	1.74E−08	IER3	20.94	0.010
EDNRB	203.30	3.39E−10	IL11	20.90	0.021
POSTN	176.93	2.90E−07	IL6	20.67	0.011
EREG	−166.10	1.02E−07	GRM1	20.19	0.013
DKK1	161.32	2.54E−09	EHF	20.08	0.016
SORL1	153.42	4.34E−10	SERPINB2	16.43	0.014
ODZ2	153.08	2.17E−07	CCL8	15.98	0.013
KYNU	−147.83	2.11E−09	ID3	−15.53	0.010

**Figure 5:**
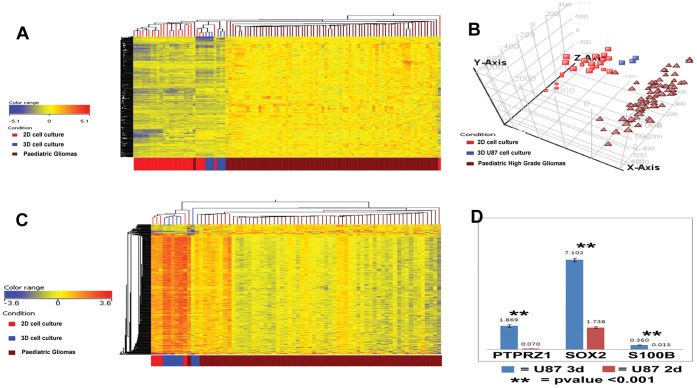
Gene and microRNA expression in RCCS aggregates resembles an intermediate phenotype between 2D cultures and primary tumors. **A –** Clustering dendrogram of significantly differentially expressed probes from Affymetrix U133 plus2 gene expression analysis between U87 and KNS42 2D cell cultures, 3D cell cultures and a cohort of pediatric high grade gliomas with 3D profiles clustering intermediate to 2D and actual tumors, also demonstrated for the U87 3D cultures in a principal components analysis (**B**). **C –** Unsupervised clustering dendrogram performed on Nanostring microRNA profiles, demonstrating 3D samples generate a profile intermediate to 2D cultures and actual tumors. **D –** Realtime PCR validation for selected genes of the gene expression array data, showing significantly elevated levels of PTPRZ1, SOX2 and S100B in 3D compared to 2D culture for U87 cells.

On comparison between 3D cultures, 2D cultures and primary tumor specimens, expression levels of numerous genes were significantly more similar between 3D and the primary tumor set than between 2D and the primary tumor set (p values <0.05). The 20 genes most differentially expressed between 2D culture and primary tumors are listed in Table 2. The expression of all bar one of these genes is more similar in 3D culture when compared to the expression level seen in primary tumors. The full list of all genes significantly differentially expressed between 2D, 3D and primary tumors is included in Table S7. Many genes already implicated in cancer and high grade glioma in particular are included in Table 2, e.g. PTPRZ1 and SOX2. Other genes have clear links to neuronal and astrocytic development e.g. TUBB2B and FABP7. In total 2604 genes had fold changes two or more times more similar to primary tumor in 3D compared to 2D. 818 genes were two or more times more similar to primary tumor in 2D compared to 3D. Ingenuity analysis of the differentially expressed genes for U87 demonstrated that the top physiological functions associated with these genes included nervous system development, embryonic development and connective tissue development. The same analysis for KNS42 suggested that tumor and tissue morphology functions are implicated the most. Genome wide profiling of the 3D cultures in general demonstrated gene expression profiles intermediate between 2D monolayer cultures of the same cell lines and a large cohort of pediatric high grade gliomas, clustering next to the actual tumors on unsupervised hierarchical clustering analysis (Figure 5). The intermediate nature of the 3D culture genetic profile is evident through principal components analysis of U87 3D culture, U87 2D culture and the tumor cohort, with the 3D culture clustering between the monolayer samples and the real tumors. The genetic changes observed in the genome wide analysis were validated on gene specific realtime-PCR for selected genes as shown in Figure 5.

**Table 2:** Differentially expressed genes amongst U87 2D/3D culture and primary HGG.

Gene	Fold change([2D]vs. [Tumor])	Fold change([RCCS]vs. [Tumor])	Ratio FC 2d:3d toprimary tumor	Correctedp-value
PTPRZ1	−491.37	−1.60	307.65	3.78E−09
GPM6B	−471.23	1.15	410.33	3.80E−13
SPARCL1	−391.25	−11.30	34.63	6.16E−08
GFAP	−364.51	−98.91	3.69	4.43E−15
TFPI2	342.55	1.06	322.65	1.79E−06
PMP2	−302.93	−16.77	18.06	9.02E−06
AQP4	−284.39	−339.70	0.84	2.76E−06
GPM6A	−242.65	−1.07	227.83	5.35E−09
SOX2	−223.37	1.56	143.57	1.01E−21
ABAT	−212.84	−37.53	5.67	2.82E−07
HSPA1A	−161.45	−42.70	3.78	2.94E−07
TUBB2B	−136.40	−1.92	71.17	4.38E−11
EREG	122.55	−1.05	116.34	5.60E−08
OLIG1	−121.24	−103.53	1.17	1.79E−05
ATP1A2	−120.92	−85.93	1.41	1.12E−05
SEPP1	−117.92	−3.44	34.26	2.19E−07
KYNU	106.24	−1.21	88.07	4.80E−10
DKK3	−105.00	1.19	88.59	8.06E−15
SLC1A2	−104.62	−78.97	1.32	4.55E−08
C13orf15	−102.58	−17.84	5.75	2.90E−06

The twenty genes most differentially expressed between 2D culture of U87 and primary HGG with the comparative fold change between RCCS culture of U87 and HGG for each gene. The ratio of the fold changes indicates greater similarity between RCCS culture and primary tumor if greater than one and greater similarity for 2D culture if less than one.

On analysis of the microRNA profile using the Nanostring platform a similar pattern of results was demonstrated. Cell lines cultured in 3D displayed a profile intermediate between actual tumors and the 2D monolayer cell line cultures. This relationship is demonstrated in the unsupervised clustering dendrogram (Figure 5). MicroRNAs most differentially expressed included miR-630 (4.2 fold increased expression in 3D cultures), miR-1308 (3.9 fold downregulated in 3D) and miR-19b (3.7 fold downregulated in 3D).

### Differential Metabolic Profiles Evident through RCCS and Monolayer Culture

**Figure 6:**
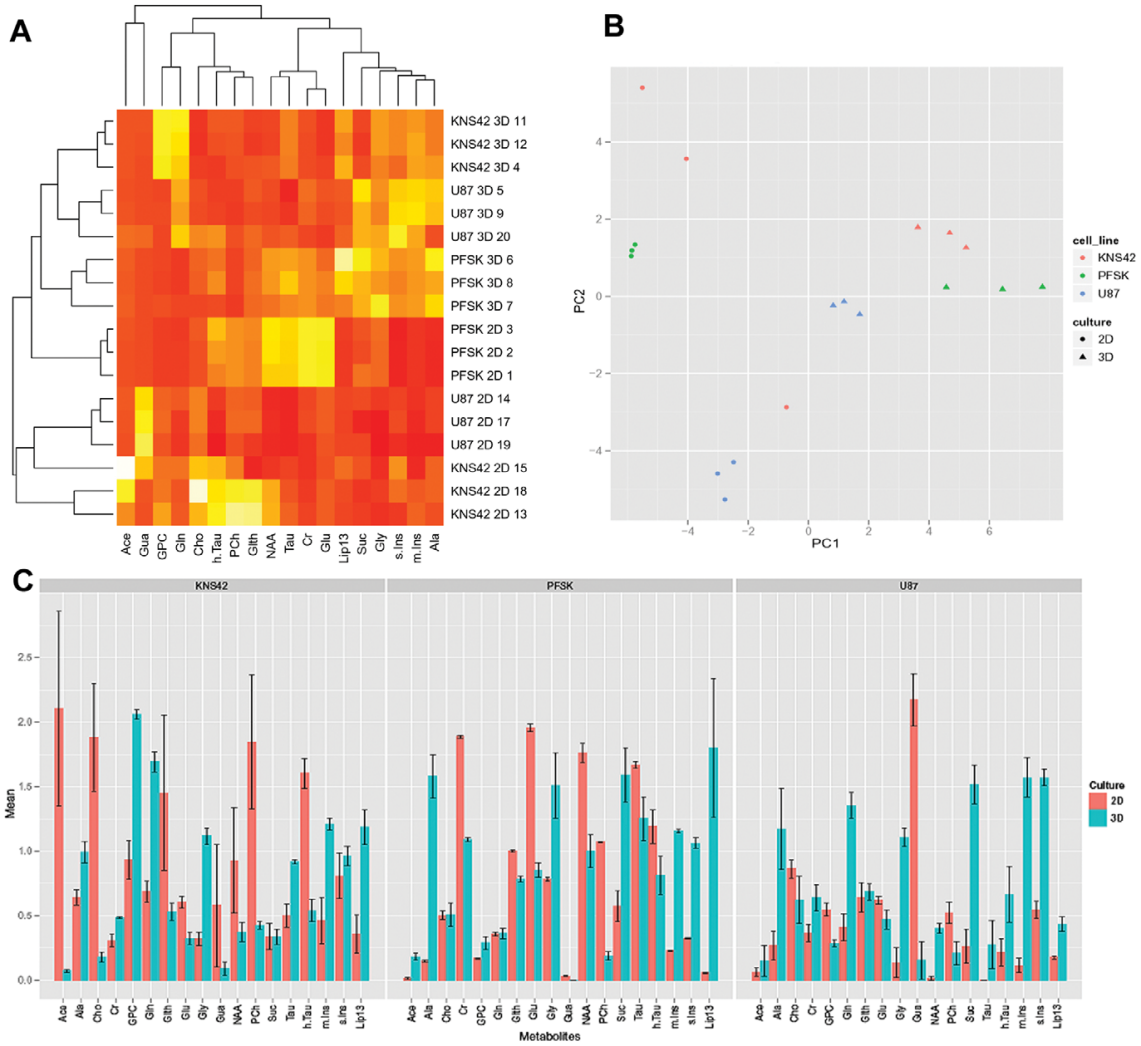
RCCS aggregates and 2D monolayers exhibit distinct metabolic profiles. **A –** Heatmap dendrogram of the HR-MAS metabolic profiles generated for KNS42, U87 and PFSK1 cell lines grown in 2D and 3D, showing clustering together of replicates grown by the same culture method with metabolites on the x-axis and samples on the y-axis. **B –** Principal components analysis of the HR-MAS data demonstrates further clear clustering by cell type and culture method. **C –** Barplot of metabolite levels for the three cell lines, comparing 2D and 3D culture averaged across three replicates for each sample with standard error bars, demonstrating significant differences for many metabolites between culture methods with consistent increases in lipids, myo-inositol and glycine and decreased phosphocholine in 3D for all cell lines.

Magnetic resonance spectroscopy reveals distinct metabolite profiles of U87, KNS42 and PFSK-1 cells grown in 2D and 3D conditions. Three replicates were prepared and analyzed to assess reproducibility. A clear clustering of the 2D and 3D samples demonstrates that the culture method represents the greatest source of influence on the metabolic profile. Subsequent levels of clustering show the close grouping of replicates from the same cell line, making this the second greatest source of influence on the metabolic profile (Figure 6 A–B). The 3D culture method shows a consistent increase in lipids, myo-inositol and glycine with a reduction in phosphocholine for each line relative to the 2D cultures (Figure 6C).

### RCCS Glioblastoma Aggregates Demonstrate Reduced Sensitivity to Histone Deacetylase (HDAC) Inhibition

**Figure 7:**
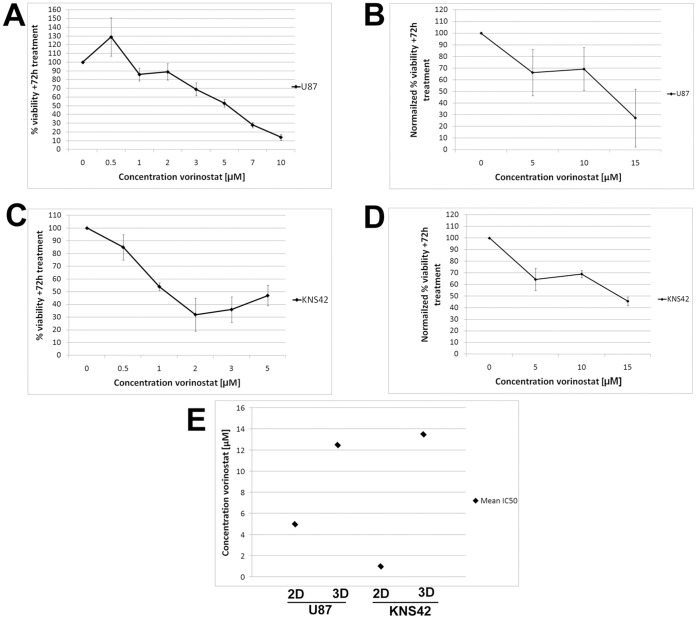
RCCS glioblastoma aggregates demonstrate reduced drug sensitivity. U87 and KNS42 glioblastoma cultures (monolayer or RCCS) were treated with Vorinostat for 72 hours prior to assessment of proliferation using the Alamar Blue assay. **A** – U87 2D cells show dose-dependent sensitivity to Vorinostat with an IC_50_ ∼5 µM. **B** – U87 aggregates generated using the RCCS are less sensitive to Vorinostat with an IC_50_ ∼12.5 µM. **C** – KNS42 2D cells show acute sensitivity to Vorinostat with an IC_50_ ∼1 µM. **D** – KNS42 aggregates generated using the RCCS are markedly less sensitive to Vorinostat in comparison with an IC_50_ ∼13.5 µM. **E** – Mean IC_50_ values calculated from A–D. Drug treatment data on 2D monolayer cultures are expressed as percentage viability relative to untreated cultures and presented as the mean of three independent experiments with standard error of mean shown. Drug treatment data on 3D aggregates are presented as the mean of two independent experiments that are normalized to Alamar Blue readings of each culture immediately prior to drug exposure with standard error of mean shown.

Aberrant HDAC expression has been linked conceptually and mechanistically to the pathogenesis of cancer due to perturbation of acetylation-deacetylation homoeostasis. As such, a number of HDAC inhibitors (HDACi) are under pre-clinical and clinical investigation as viable anticancer agents [50], [51]. Promising *in vitro* results has lead to several brain tumor clinical trials using HDACi, with a Vorinostat phase II trial in adult recurrent glioblastoma multiforme showing anti-cancer activity [52] and a Vorinostat phase I trial in synergy with 13 cis-retinoic acid, recently completed for pediatric medulloblastoma and CNS primitive neuroectodermal tumors [53]. We hypothesized that RCCS derived aggregates would render brain tumor cells less sensitive to Vorinostat compared to 2D monolayers as proof-of-concept of reduced drug sensitivity within the 3D tumor microenvironment. U87 and KNS42 glioblastoma monolayer cells were exposed to an acute Vorinostat dose (0.5 µM–10.0 µM) for three days and assessed for metabolic activity thereafter. Both cell lines broadly exhibited dose-dependent sensitivity to Vorinostat with an IC_50_ of 5 µM and 1 µM for U87 and KNS42 respectively (Figure 7A and C). Based upon our prediction of reduced drug sensitivity in 3D culture, we exposed one week 3D U87 and KNS42 cultures to Vorinostat concentrations of 5 µM, 10 µM and 15 µM. To control for stochastic variations with respect to aggregate size after one week in RCCS culture, we normalized metabolic activity after 72 hours drug treatment to metabolic activity after 0 hours (i.e. point at which macroscopic aggregates had formed and prior to drug exposure). For both untreated and drug treated samples, the same aggregate was assessed at 0 and 72 hours for metabolic activity. Due to an observed reduction in viability in the 3D KNS42 untreated culture (Figure 7D), most likely due to necrosis in the aggregate core being greater than proliferation in the rest of the aggregate, KNS42 3D drug treated cultures were further normalized to the proportion of KNS42 untreated cells that were viable only. Both high grade glioma cell lines demonstrated significantly reduced sensitivity to Vorinostat with IC_50_ of 12.5 µM and 13.5 µM for U87 and KNS42 respectively (p value <0.05) (Figure 7B and D). This amounts to a 2.5 fold reduction in Vorinostat sensitivity in U87 and a 13.5 fold reduction in KNS42 when assaying in 3D culture conditions (Figure 7E).

### Primary Explant Culture in the RCCS Retains Features of the Primary Brain Tumor

**Figure 8:**
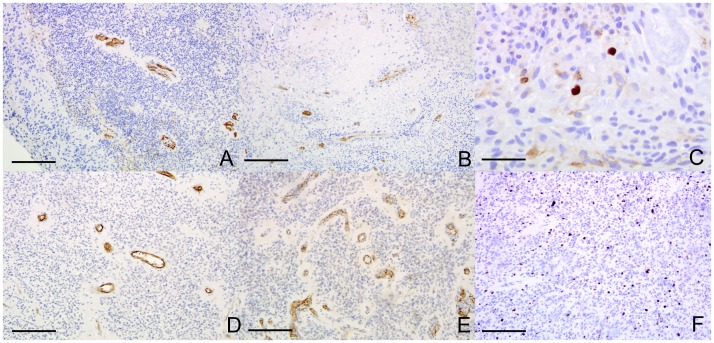
Fresh tissue explant culture in the RCCS maintains primary tumor phenotypes. **A – C** Three week primary explant cultures of pediatric anaplastic ependymoma stained immunohistochemically for CD105, CD31 and Ki67 respectively, demonstrating rim/core division with sparsely cellular core and positivity for vessel markers and Ki67. **D – F** Immunohistochemistry against CD105, CD31 and Ki67 respectively on primary tumor sections from the same pediatric anaplastic ependymoma from which the explant was derived in A–C for comparison. Scale bars 25 µm in C, 100 µm in all others.

Primary glioma tumor explants were taken directly from the operating theatre and placed into the RCCS. Culture in standard medium was maintained for 3 weeks, with the gross structural integrity of the explant preserved. Explants began to develop a necrotic core and viable rim in a similar fashion to cell line derived aggregates. Additional features of internal histology were preserved from the primary tumor, including heterogeneity and cells staining positive for endothelial markers in blood vessel walls. Explants demonstrated positive Ki67 staining after 3 weeks of culture but at lower levels than the proliferative areas of actual primary tumor (Figure 8). A conventional 2D monolayer cell line was derived from an adult GBM tumor and comparison with a sample of the same tumor cultured for three weeks as an explant made using the ECM real-time-PCR array. There was significant variation in gene expression between the two samples with genes such as ITGB2, KAL1, MMP 8/9/12, NCAM1 and SPP1 upregulated significantly in the explant and THBS1, CTGF, COL1A1 and COL14A1 expressed at significantly lower levels (P values<0.05) in the explant (data not shown).

## Discussion

Although the clinical trial will remain the ultimate testing ground for evaluation of novel next-generation anti-cancer therapies, these are expensive with a myriad of logistical, regulatory and ethical considerations. Therefore, representative preclinical model systems that are both laboratory and computational based, need to provide an intermediate testing ground for experimental agents. However the long history of use of 2D cell panels such as the NCI-60 has often proved relatively poor at predicting both *in vivo* animal model and early phase clinical trial efficacy and toxicity. The limitations of relating 2D cell panels to clinical tumors is due to a tumor microenvironment far removed from the physiological setting, inaccurate drug uptake and altered pharmacokinetics/pharmacodynamics of the selected drug(s). The present study provides a comprehensive characterization of the RCCS as a method of cancer cell culture to generate a tumor niche more physiologically relevant than 2D culture and drug response more representative of clinical high grade brain tumor drug response, than conventional 2D cell panels.

It is increasingly clear that cancer is a process involving complex interactions between tumor cells and the surrounding microenvironment, including non-neoplastic cells, ECM and environmental conditions such as oxygen tension [54], [55]. Critically, these conditions vary within different regions of high grade brain tumors with a characteristically hypoxic necrotic core; an intermediate zone where cells survive but under hypoxic strain and the periphery of the tumor where cancer cells invade into relatively well oxygenated brain. Models have been proposed whereby this heterogeneity of conditions gives rise to different sub-populations of tumor cells which fulfill different functions and niches within the tumor as a whole. Much interest has focused on the immediate vicinity of small tumor associated blood vessels within the brain, termed the microvascular niche, as a possible favorable habitat for the stem-like cells which may be critical in tumor propagation and resistance to treatment [56], [57]. It may be that different areas of the tumor may favor other sub-clone populations such as proliferative or invasive cells. Monolayer cell culture creates a relatively homogeneous population of cells that are all subject to the same selection pressures. Heterogeneity between different areas within the same tumor has long been recognized histologically, but has also now been demonstrated molecularly with different driving genes amplified in different areas of a glioblastoma [6]. This heterogeneity may partially explain the failure of targeted therapies, with only a subset of tumor cells being potentially sensitive to a particular compound. The RCCS model recapitulates heterogeneity histologically, allowing more representative drug testing against the entirety of a tumor rather than solely the proliferative fraction present in a 2D monolayer.

Monolayer cell culture typically takes place in room air (21% oxygen content) with supra-physiological concentrations of glucose and other metabolites in the feed medium. The normal brain oxygen saturation is around 7% with tumor regions ranging from 5% to less than 1% [58]. Cell culture can be performed in a hypoxic chamber, though this still does not replicate the gradient in oxygen concentration seen in tumors *in vivo*. Previous studies have demonstrated that the oxygen concentration to which cells are exposed is of critical importance in defining the behavior of those cells in high grade brain tumors [59], [60], [61]. This may have direct therapeutic implications in areas such as the derivation of dendritic cell based anti-cancer vaccines. It also seems that the gene expression profile of tumors is affected by region, with biopsies taken from more ischemic/necrotic regions displaying a more mesenchymal genotype [62]. The behavior of cancer stem-like cells is also known to be profoundly impacted by their environmental oxygen levels [63], [64], [65]. Many processes involved in the development of CNS tumors are also by definition 3D such as angiogenesis and interaction with the ECM. Again, these processes are not re-capitulated in 2D culture which is a highly reductionist model. We have demonstrated that an oxygen gradient exists across the RCCS aggregates replicating a key feature of the environmental conditions to which brain tumors are exposed. This seems likely to have implications for cell behavior within the culture model, crucially replicating the exposure of different areas of the cell population to different conditions within the same tumor. Members of our group have previously demonstrated similar findings in breast cancer where the hypoxic, relatively harsh micro-environment seems to select for a more aggressive metastatic phenotype [66].

*In vivo* animal models typically consist of xenotransplantation of a human cancer cell line into an immunocompromised rodent host. Orthotopic grafting replicates many facets of the CNS environment in which human tumors develop and some processes such as development of a vascular network. However, these models all have a rodent host as their basis and the disappointing record of therapeutic agents taken into phase I/II trials following animal studies demonstrates how specific tumor/host environment interactions are critical in governing tumor behavior. It is as yet unclear whether, for example, interactions between tumor and endothelial cells are species specific. The immune system (known to have a key role in human brain tumors) in these animals also behaves very differently to that in a normal human, further reducing the strength of animal models. Animal experiments have other drawbacks such as their cost and the ethics of utilizing large numbers of animals in research with an increasing pressure to reduce the use of animals in research and drug development.

Other 3D models have been used to try and overcome these issues and the most widely used of these in cancer and stem cell research is the non-adherent neurosphere culture. It is well recognized that this culture method increases the proportion of cells displaying stem-like properties and markers [67]. Other advanced culture methods used to model tumor growth include the hanging drop method (creating similar entities to neurospheres) [68], hollow fibers [69], microfluidic chambers and multicellular layer constructs [70]. The latter three methods still utilize support membranes or fibers which provide an artificial physical contact point for cells and do not replicate true unrestricted growth in 3D. Neurospheres range in size from around 50 µm to a maximum of around 300 µm in diameter (compared to 1–9 mm for RCCS aggregates). At their largest size they begin to exhibit a necrotic core [71] in a similar fashion to RCCS aggregates, though the effect is significantly less pronounced. Neurosphere culture generally involves culture media that differ from the standard preparations used for monolayer culture; for example neurosphere medium is usually serum free and supplemented with epidermal growth factor and basic fibroblast growth factor. The RCCS aggregates are formed in the same medium as used for 2D cell culture, thus removing the influence of media composition on comparisons between RCCS and 2D culture using the same cell line. The recent demonstration of a 3D culture system with automated and quantitative analysis, similarly generates tumor spheroids with a size range of 300–500 µm in diameter [24]. Although matrix invasion and angiogenic differentiation was induced when tumor cells were co-cultured with embryoid bodies, we believe that the ∼10-fold larger RCCS aggregates display more acute cellular heterogeneity with clearly delineated proliferating rim, necrotic core and peri-necrotic rim with direct observation of hypoxic stress and senescent cells in this region. Indeed we have also shown that a proportion of RCCS brain tumor aggregates exhibit a vasculogenic phenotype and angiogenic gene expression profile in the absence of any form of co-culture, a phenomenon termed ‘vasculogenic mimicry’ (Smith et al, manuscript in preparation). Moreover as the immediate tumor microenvironment is generated by the secretion of endogenous ECM rather than exogenous and artificial substrates, the resulting paracrine signaling and resulting global gene expression changes in the RCCS may be more informative than other 3D methods. In support of the importance of cancer cell-ECM interactions with regards to mimicking physiological aspects of tumor biology, Loessner and colleagues describe a 3D culture consisting of a sophisticated synthetic hydrogel matrix with incorporated biomimetic features. This method permitted exposure to similar biochemical and biomechanical stimuli in all directions due a homogenous dispersion of ovarian tumor cells. Importantly, increasing matrix stiffness inhibited spheroid growth resulting in a more compact spheroid. A recent high-throughput automated 3D culture system based upon microfluidic channels similarly permits the generation of homogenous and densely packed tumor cell aggregates in a reproducible manner, resulting in increased drug resistance compared to 2D cultures. These findings implicate cell density, cell-cell interactions and cell-ECM interactions as contributing mechanism by which the proliferation rate of cells in 3D may be relatively slower than corresponding 2D cultures and additionally why 3D cultures may be relatively more drug-resistant [72], [73]. We have profiled the same cell lines after culture in 2D and 3D RCCS, demonstrating statistically significant differences in the expression levels of many key genes known to be involved in the neoplastic process. Profound changes take place between 2D and 3D in the expression of genes involved in areas such as ECM, cell proliferation and stem-like characteristics. These changes alter the overall genetic profile of the sample, rendering them significantly more similar to the profiles exhibited by our cohort of primary pediatric high grade gliomas, though there are clearly still differences between 3D cultures and clinical tumors. Investigation of agents targeting specific molecular pathways is again likely to be more efficacious in model systems such as the RCCS which more closely replicates gene expression and protein activity levels observed in clinical tumors. Sensitivity to drugs will depend upon the exact expression levels of many genes and it is clear that radically different and less meaningful results are likely to be achieved in drug testing using 2D compared to 3D culture.

Similar results are observed when considering the metabolic profiles generated by the *in vitro* MRS analysis performed. Culture method was found to be the most important factor in generating the metabolic profile and samples cluster accordingly. Lipids are consistently increased in 3D compared to 2D culture, which typically represents increased levels of cell death, accurately reflecting the changes observed in the histology of aggregates with their necrotic centers. Elevated lipid peaks are also typically observed in necrotic regions of actual high grade brain tumors, especially glioblastomas [74]. The decreased phosphocholine observed in 3D culture is consistent with slower growing cells such as observed *in vivo* after chemotherapy [75] which provides metabolic corroboration of the proliferation rates observed in our study. Moreover a recent study provides evidence that homoeostasis in 3D tumor aggregates is achieved through a balance between cell proliferation, growth arrest and cell death [76], supporting the recapitulation of heterogeneous tumor sub-populations within 3D systems as described here. Increased myo-inositol and glycine may correspond to higher grade behaviors within tumors [77] and their elevation in 3D compared to 2D culture is intriguing and warrants further investigation.

Resistance to chemotherapeutic drugs has been shown to differ significantly for cell lines grown *in vivo*, in neurospheres or in monolayers [78], [79], [80], [81], [82]. Resistance is generally highest *in vivo* and lowest in 2D, varying by at least two orders of magnitude. Through exposure to the histone deacetylase inhibitor Vorinostat, our cultures in the RCCS confirm the markedly enhanced resistance conferred by 3D culture (2.5 fold and 13.5 fold for U87 and KNS42 cells respectively) which has significant implications for pre-clinical drug testing strategies and dosage regimes to be utilized *in vivo*. A significant over-estimate of agent potency is likely using solely 2D data. Even when considering Vorinostat exposure in brain tumor neurosphere cultures (IC_50_ 0.5 µM) that enhance for cancer stem/progenitor cells [83], the RCCS aggregates are relatively more resistant indicating that the RCCS may provide a better *in vitro* system to evaluate agents targeting stem/progenitor pathways. A key feature of RCCS culture is the creation of a tissue barrier between agents applied exogenously and cells deep within the aggregate. This creates the necessity for agents to penetrate significant depths into tissue, replicating *in vivo* conditions. This may be further compounded by the formation of cell-secreted ECM within aggregates that may limit drug penetrance and enhance integrin-mediated pro-survival signaling [73], [84]. Such considerations are of key importance when considering the interaction between drugs and also the genetic and epigenetic profiles emerging from various methods of culture. The more resistant phenotype of RCCS aggregates may be directly associated with a lack of homogenous drug exposure within the culture. However, our results do not exclude the possibility that the RCCS microenvironment facilitates the creation of a niche that enriches for and/or harbors brain tumor sub-populations that are intrinsically resistant to Vorinostat. It is likely that both phenomena contribute to the relatively more resistant phenotype in 3D compared to 2D cultures. The increased resistance shown by aggregates containing areas of hypoxic cells may be consistent with enrichment of glioma stem-like cells in hypoxic and vascular niches that may be intrinsically resistant to chemotherapy [15], [57], [63].

Importantly, our findings contrast to a recent study assessing the efficacy of Vorinostat against ependymoma brain tumor stem cells propagated using the neurosphere assay. Witt and colleagues report an IC_50_ value of 0.78 µM when ependymoma neurosphere cultures were treated to a clinically achievable concentration range of Vorinostat and abolition of neurosphere initiating capacity (i.e. self-renewal capacity) at an IC_50_ of 0.5 µM [83]. Based solely on IC_50_ values, the ependymoma neurosphere cultures were 16–17 fold more sensitive than our RCCS aggregate cultures, supporting the notion that the RCCS tumor microenvironment induces greater intrinsic tumor cell resistance and/or better reflects difficulties in achieving effective drug dose penetration *in vivo*. However, we cannot exclude the possibility that differences in Vorinostat efficacy is at least in part due to the different cell types and lines used in each study. In a similar finding, treatment of GBM neurospheres with the telomerase antagonist Imetelstat, led to an IC_50_ value of 2 µM after prolonged 4-week exposure [85] enforcing the hypothesis that neurospheres are relatively more sensitive than RCCS aggregates. IC_50_ values detected in the micromolar range in our study highlight a caveat of standard 2D drug screens where an agent is typically discarded if its potency is not in the nanomolar range. Conversely there are instances of clinically failed anti-cancer compounds with nanomolar potency *in vitro* [86]. We propose introducing the RCCS as a second tier preclinical efficacy/toxicity testing tool, whereby targets forwarded from 2D drug screens will be further scrutinized in the RCCS prior to the uptake of lead compounds for *in vivo* trials. Furthermore it will also be important to consider sequential exposure of RCCS aggregates to drug combinations as enhanced doxorubicin accumulation and toxicity has recently been shown when tumor spheroids were pre-treated with mitoxantrone or paclitaxel [87].

We have also demonstrated that the RCCS can be used as a system for maintaining viable explants of primary tumor direct from surgery for at least 3 weeks, a distinct advantage over alternative 3D spheroid culture systems [24], [28], [29], [30]. Viable dividing cells are maintained and internal architecture is preserved including the presence of endothelial cells. The centre of explants develops necrotic areas in a similar fashion to cultured aggregates, indicating a similar tolerance level to hypoxia. Further investigations will encompass more clinically-relevant drug testing against these primary explants which may allow prediction of the response of the tumor in the patient to the same agent. Array real-time-PCR shows clear differences in gene expression between early passage cell lines derived from the tumor and subsequently cultured in 2D and tumor explants cultured in the RCCS.

### Conclusions

Our comprehensive characterization demonstrates that 3D RCCS culture of high grade brain tumor cells has profound effects on the genetic, epigenetic and metabolic profiles of cultured cells, with these cells residing as an intermediate phenotype between that of 2D cultures and primary tumors. We have additionally shown that RCCS GBM aggregates are relatively more resistant to the HDACi Vorinostat when compared to 2D monolayer cultures and likely represents a more faithful drug response observed in the clinical setting. The RCCS provides a platform for undertaking a variety of investigations in the Cancer and Neuroscience fields and our findings encourage broad utility of this *in vitro* experimental system to interrogate normal and dysregulated neural cell behavior and to evaluate candidate therapeutic compounds. We would anticipate that RCCS cultures of other cell types and primary tissue would behave very differently compared to 2D culture methods and the RCCS could have wider applicability to testing therapies against other neuronal and glial cell systems as well as wider applicability in the cancer field. Treatment advances for brain tumors and other neurological disorders will be based on a thorough understanding of the molecular pathways involved with the pathologies in question. It seems imperative that we should model diseases and test therapies using *in vitro* systems that approximate the *in vivo* situation as closely as possible to give the best chances of therapies successful *in vitro* maintaining their effectiveness in animal or clinical trials. The RCCS offers a means of achieving key facets of gene expression and metabolism using an *in vitro* model and presents an opportunity to advance our understanding of how the heterogeneity and differing environmental pressures within a tumor contribute to its overall biology and response to therapeutic agents.

## Supporting Information

Figure S1 **Upregulation of ICAM-1 in 3D culture.** Immunohistochemistry of ICAM-1 confirms the upregulated expression of ICAM-1 in (A) 3D relative to (B) 2D culture of KNS42 cells and validates the ECM array data in Figure 4.
(TIF)

Table S1 **ECM genes PCR array.**
(XLS)

Table S2 **PCR primer data.**
(XLS)

Table S3 **Nanostring probes and targets.**
(XLS)

Table S4 **Full list genes differentially expressed ECM arrays.**
(XLS)

Table S5 **Full list genes differentially expressed U87 3d vs. 2d.**
(XLS)

Table S6 **Full list genes differentially expressed KNS42 3d vs. 2d.**
(XLS)

Table S7 **Full list genes differentially expressed U87 tumor vs. 2d vs. 3d.**
(XLS)
