# Supplementary figures and images for: Recapitulation of Tumor Heterogeneity and Molecular Signatures in a 3D Brain Cancer Model with Decreased Sensitivity to Histone Deacetylase Inhibition
Source: PLoS One. 2012 Dec 18;7(12):e52335. doi: 10.1371/journal.pone.0052335 (PMC3525561; doi:10.1371/journal.pone.0052335)

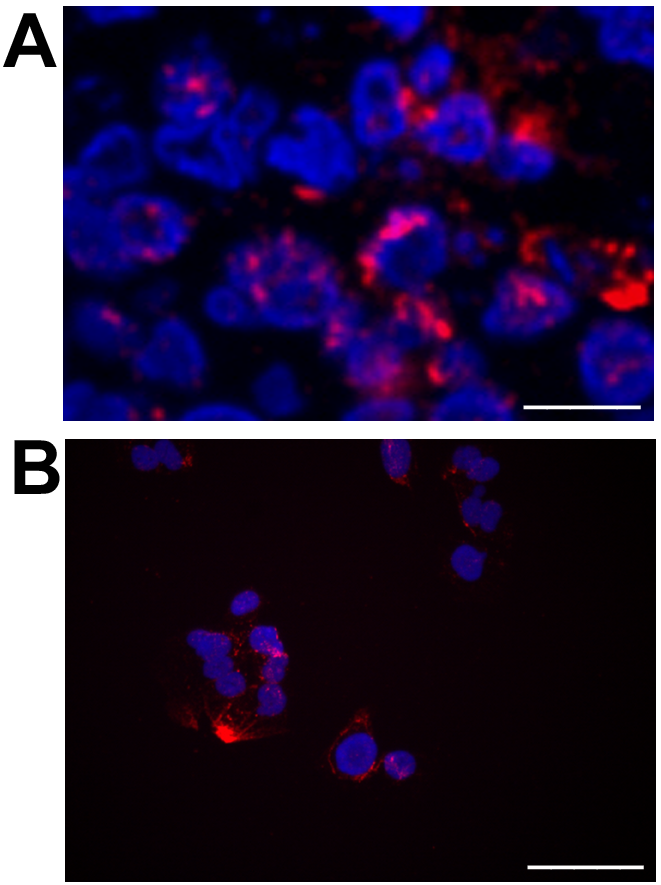

Supplement: Figure S1 — Upregulation of ICAM-1 in 3D culture. Immunohistochemistry of ICAM-1 confirms the upregulated expression of ICAM-1 in (A) 3D relative to (B) 2D culture of KNS42 cells and validates the ECM array data in Figure 4. (TIF) [file pone.0052335.s001.tif]
